# Molecular Mechanisms Underlying the Synergistic Regulation of Glucose and Clay Minerals on Polyphenol-Maillard Mediated Abiotic Humification

**DOI:** 10.3390/molecules31071127

**Published:** 2026-03-29

**Authors:** Yanyan Liu, Haoyu Gao, Tao Fu, Mingshuo Wang, Houfu Chen, Shuai Wang

**Affiliations:** 1 College of Agriculture, Jilin Agricultural Science and Technology College, Jilin 132101, China; 18632442685@163.com (Y.L.); 17535003652@163.com (H.G.); wangmingshuo@jlnku.edu.cn (M.W.); 2 Jiaohe Agricultural Technology Extension Station, Jilin 132500, China; ft13943205035@163.com; 3 Department of Chemical Engineering, University of Waterloo, Waterloo, ON N2L 3G1, Canada; h534chen@uwaterloo.ca

**Keywords:** abiotic humification, glucose, kaolinite, montmorillonite, humic-like substances, humic-like acid

## Abstract

The synergistic effects of glucose (Glu) concentration and clay mineral type (kaolinite [Kao], montmorillonite [Mon]) on abiotic humification via the polyphenol-Maillard reaction remain poorly understood. To address these scientific challenges, a series of controlled, sterile batch experiments was conducted. Specifically, a glucose concentration gradient (0, 0.03, 0.06, 0.12, and 0.24 mol/L) was established; Kao and Mon were separately introduced as mineral catalysts; and the Maillard reaction was facilitated in the presence of catechol and glycine under strictly abiotic conditions to preclude any potential biological interference. Comprehensive analyses were performed on the reaction products—namely, the supernatant and the dark-brown residue generated during the reaction process. These analyses included: the E_4_/E_6_ ratio and total organic carbon (TOC) content of the supernatant; the carbon-based ratio of humic-like acid to fulvic-like acid (C_HLA_/C_FLA_); and the structural characteristics of humic-like acid (HLA) isolated from the dark-brown residue. Results showed dynamic E_4_/E_6_ ratio and TOC changes in the supernatant were accurately described by the Logistic function. Kao favored soluble organic C accumulation and enhanced retention of early-stage, low-molecular-weight intermediates in the dark-brown residue, while Mon promoted humic-like substances (HLS) polymerization and aromatic condensation. FTIR spectroscopy analysis identified optimal Glu thresholds for maximal HLS formation—0.03 mol/L for Kao and 0.06 mol/L for Mon—indicating non-linear, rather than monotonic, dependence on Glu dosage. Comparative pre- and post-reaction Fourier-transform infrared (FTIR) spectroscopy further demonstrated that Mon, owing to Mg–OH octahedral sites arising from isomorphic substitution, formed more stable Cat chelates than Kao. These chelates effectively stabilized surface-bound hydroxyl-associated water molecules and modulated the electron cloud distribution around Si–O bonds. Collectively, this study clarified the dual regulatory role of Glu concentration and clay mineral identity in abiotic humification pathways, advanced mechanistic understanding of clay mineral-mediated polyphenol-Maillard reactions, and established a scientific foundation for optimizing humification efficiency in both engineered and natural systems.

## 1. Introduction

Abiotic humification represents a key geochemical process contributing to the formation of humic-like substances (HLS) in terrestrial and aquatic ecosystems, particularly in extreme environments with limited biological activity [1]. As the most widely distributed reducing sugar in soil environments, glucose (Glu) not only serves as a C source for humification but also participates in the Maillard reaction alongside amino acids and polyphenol precursors, thereby influencing the composition and structure of the resulting HLS [2]. Meanwhile, clay minerals such as kaolinite (Kao) and montmorillonite (Mon), which function as essential carriers of soil organic matter (SOM), mediate abiotic humification via adsorption and catalytic processes due to their unique structural properties [3]. Elucidating the synergistic effects of Glu concentration and clay mineral types on abiotic humification is critical for understanding soil C sequestration and nutrient cycling, representing a pressing scientific challenge in the fields of soil chemistry and environmental geochemistry.

The process and product characteristics of abiotic humification, as a key pathway for soil C sequestration, have become a research hotspot, especially in the aspects of clay mineral mediation, C source ratio regulation, and specific precursor system transformation. Regarding the mediating role of clay minerals in abiotic humification, Heryanto et al. clearly demonstrated that Mon and Kao, due to their structural differences, had significant distinctions in the adsorption and catalytic polymerization of humification precursors. Mon, with its interlayer expansibility and high cation exchange capacity, was more likely to promote the formation of stable humic-like acid (HLA) through chemical bonding. Although Kao had a more rigid structure, the non-parallel distribution of charges in its crystal structure could enhance the interaction with precursors, providing a mineral selection basis for the regulation of specific precursor systems’ humification [4]. Almendros and González-Pérez. further confirmed that the synergistic effect of clay minerals and mineral oxides (such as MnO_2_) could accelerate the polyphenol-Maillard reaction, especially in specific precursor systems like catechol (Cat)-glycine (Gly), enhancing the structural stability of HLA through catalytic oxidative polymerization, further enriching the understanding of the mechanism of clay mineral-mediated abiotic humification [5]. He et al.’s research on clay-based modified materials further confirmed that the functional groups and ions on the surface of clay minerals could synergistically optimize the efficiency of abiotic humification, providing important references for the application of Kao and Mon. The regulatory effect of C source ratio on abiotic humification has been widely verified [6]. Sun et al. found in their artificial regulation of humification research that in mineral catalytic systems (such as Fe/Mn oxides), the type and ratio of C sources could influence the aromatization degree and functional group composition of HLA by regulating the polymerization path of precursors, providing a practical reference for the directional regulation of Glu ratio [7]. Li et al. further confirmed in their sterile system research that the concentration of C sources (Gly) could significantly affect the characteristics of abiotic humification products, changing the aromatic condensation degree and oxygen-containing functional group content of HLS by regulating the polymerization efficiency of precursors, complementing the research of Sun et al. [8]. Zhou et al.’s research on functional biochar revealed that in the Glu and biochar composite system, C sources could provide sufficient intermediates for the polymerization of precursors by participating in the polyphenol-Maillard reaction, thereby influencing the structural stability of HLA [9]. In terms of the catalytic mechanism and product analysis of abiotic humification, related studies have provided important support for this article. Long et al.’s research on composite mineral catalysis clearly stated that the core of abiotic humification lay in the catalytic effect of minerals on the polyphenol-Maillard reaction, which could accelerate the oxidative polymerization of precursors and enhance the structural stability of HLA [10]; Song et al.’s analysis of the mechanism of phenolic humification confirmed that the oxidative polymerization of phenolic precursors like Cat was the key to the formation of the aromatic structure of HLA [11]. Sarlaki et al.’s research on mild reaction systems further verified the feasibility of abiotic transformation of phenolic and amino acid precursors, providing a reference for experimental condition design [12]. The development of structural analysis techniques for HLA has made it possible to quantitatively study the mechanism. Park et al.’s review of two-dimensional correlation spectroscopy (2D-COS) technology could precisely track the dynamic evolution sequence and binding sites of functional groups during the humification process, providing technical support for the structural characterization of HLA [13]. Furthermore, the studies by Fragouli et al. and Zhang et al. [14,15], respectively, starting from the environmental functions of humus and the transformation pathways of its precursors, had confirmed that the structural characteristics of abiotic humification products directly determined their C sequestration efficiency, further highlighting the significance of regulating the composition and structure of HLS.

Although existing studies have initially clarified the regulatory roles of clay minerals and precursors in abiotic humification, the cooperative mechanism of Glu proportion and Kao or Mon, especially how the structural differences in Kao or Mon affect the composition of HLS through the Glu-mediated pathway, have not been fully elucidated. Based on this, this study used Cat and Gly as model humification precursors and introduced varying concentrations of Gly. Kao and Mon were selected as representative clay mineral mediators to facilitate the Maillard reaction. Among common soil clay minerals, Kao and Mon were specifically selected for this study due to their distinct surface characteristics at the experimental pH of 8.0. Kao exhibits a low cation exchange capacity (CEC), weak surface charge density, and relatively small specific surface area, while Mon possesses high CEC, strong negative surface charge, and a large specific surface area with expandable interlayer spaces and these differences that are expected to induce divergent adsorption and catalytic behaviors toward humification precursors. A comprehensive suite of analytical indicators—including the E_4_/E_6_ absorbance ratio, total organic carbon (TOC), C content of humic-like acid (C_HLA_), C_HLA_-to-fulvic-like acid carbon ratio (C_HLA_/C_FLA_), Fourier transform infrared (FTIR) spectroscopy, and atomic molar ratios (C/N, O/C, and H/C) to investigate the effects of different concentrations of Glu on the abiotic formation of HLS catalyzed by Kao or Mon. The aim was to clarify the synergistic interplay between Glu proportion and clay mineral type, deepen the mechanistic understanding of the optimization of abiotic humification efficiency, thereby revealing the abiotic-driven pathways of HLS formation.

## 2. Results

### 2.1. E_4_/E_6_ Ratio and Total Organic Carbon (TOC) Content in the Supernatant

**Figure 1:**
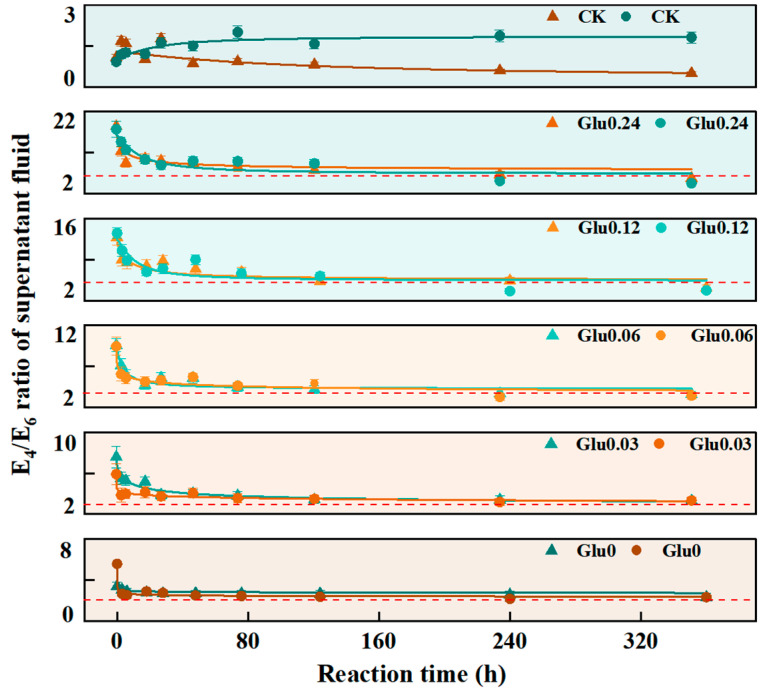
Variation in the E_4_/E_6_ ratio in the supernatant during the polyphenol-Maillard reaction. Note: A glucose concentration gradient was established at 0, 0.03, 0.06, 0.12, and 0.24 mol/L, with corresponding treatment groups labeled as Glu0, Glu0.03, Glu0.06, Glu0.12, and Glu0.24, respectively. The CK consisted solely 2 g of kaolinite or montmorillonite in phosphate buffer, without the addition of glucose, catechol, and glycine. The triangle symbol denoted Kao, while the circle represented Mon. In the scatter plots of Figure 2, Figure 3 and Figure 4, as well as the radial bar chart of Figure 5, the error bars (or error bands) indicated the standard deviation for each data point (*n* = 3). The red dash-dot line indicated the E_4_/E_6_ threshold of 3.0, a critical value for aromatic condensation of humic-like substances. All experiments were conducted over a 360 h timeline.

The E_4_/E_6_ ratio was a reliable indicator of the condensation degree of aromatic compounds and the extent of humification, a higher ratio indicated lower molecular weight and simpler structure of HLS, whereas a lower ratio suggested higher molecular weight and enhanced aromaticity [16]. As presented in Figure 1 and Table 1, during the Maillard reaction-driven abiotic humification process, the temporal variation in the E_4_/E_6_ ratio was well described by the logistic function:

(1) $$y_{1}\;=\;[A_{2}\;+\;(A_{1}-A_{2})]/[1\;+\;(x/x_{0}{)}^{p}]$$

with high model fitting accuracy, indicating robustness and reliability of the results. In this fitting equation, *y*_1_, *A*_1_ denoted the steady-state E_4_/E_6_ ratio—the asymptotic value reached in the late reaction stage—reflecting the molecular characteristics of the final HLS products; *A*_2_ denoted the initial E_4_/E_6_ ratio, representing the maximum value at the onset of the reaction and corresponding to low-molecular-weight intermediates formed during the early phase of the Maillard reaction. In the Kao system, increasing Glu concentration led to an increase in *A*_1_ from 2.57688 to 7.45618 (a rise of 189.3%) and in *A*_2_ from 3.28823 to 18.22455 (an increase of 454.2%). In the Mon system, *A*_1_ increased from 2.25521 to 5.67417 (by 151.5%), and *A*_2_ rose from 4.99665 to 16.56004 (by 231.4%). In the Kao system, the increments of *A*_1_ and *A*_2_ were significantly higher than those in the Mon system. Specifically, the growth rate of *A*_1_ was 37.8 percentage points greater, while that of *A*_2_ increased by 222.8 percentage points. Over the 360 h incubation period, all Glu-amended treatments exhibited a continuous decline in the E_4_/E_6_ ratio, with a generally greater reduction observed in the Mon system compared to the Kao system, suggesting that Mon exerted a stronger catalytic effect on the condensation of aromatic structures. Notably, all Glu-amended treatments in both Kao and Mon systems converged to values near the E_4_/E_6_ threshold at 360 h, demonstrating that regardless of initial Glu concentration, the aromatic condensation degree of HLS ultimately reached a consistent stable state.

**Figure 2:**
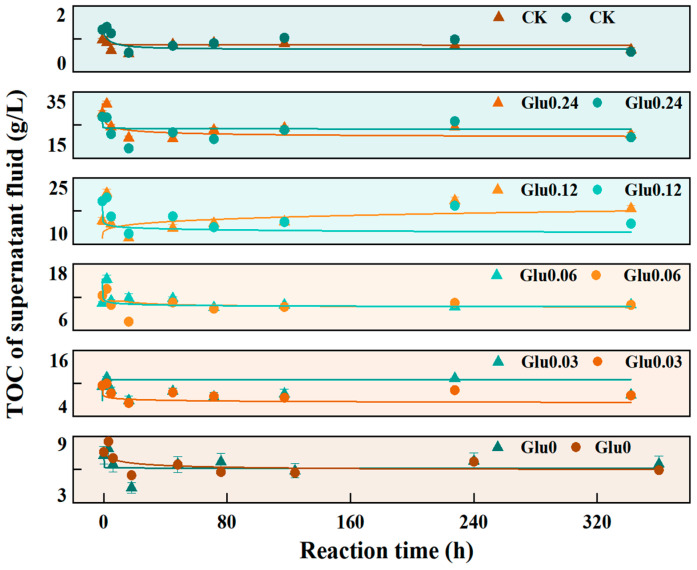
Variation in the TOC content in the supernatant during the polyphenol-Maillard reaction.

TOC content was a key indicator of organic C accumulation in the supernatant and was directly linked to the formation and transformation efficiency of HLA during the Maillard reaction [2]. As was shown in Figure 2 and Table 2. In this fitting equation:

(2) $$y_{2}\;=\;A_{\min}\;+\;(A_{\max}\;-\;A_{\min})/[1\;+\;(x_{0}/x{)}^{h}{]}^{s}$$

*y*_2_, *A_min_* represented the steady-state TOC value, reflecting the residual soluble C level upon completion of humification; *A_max_* denoted the maximum TOC achieved during the reaction period, corresponding to the peak phase of C precursor conversion; and *s* was the rate parameter that characterized the kinetic intensity of TOC change. Model fitting yielded high coefficients of determination (R^2^), ranging from 0.80175 to 0.99978, demonstrating the model’s robustness in capturing the C transformation dynamics. With increasing Glu concentration from 0 to 0.24 mol/L, both *A_min_* and *A_max_* exhibited a consistent upward trend in both Kao and Mon systems. In the Kao system, *A_min_* increased from 4.3517 to 20.8921 (a 379.9% rise), while *A_max_* rose from 7.8902 to 31.3766 (a 297.7% increase). In the Mon system, *A_min_* increased from 5.8942 to 21.56114 (by 265.7%), and *A_max_* increased from 7.75833 to 26.85043 (by 168.3%). Regarding the rate parameter *s*, under most Glu concentrations, the *s* value was lower in the Kao system than in the Mon system, except at 0.24 mol/L, where it became higher in the Kao system. In most treatments with Glu supplementation, the TOC concentration rapidly peaked at 3 h of reaction, followed by a fluctuating decline until the conclusion of the 360 h reaction period.

### 2.2. E_4_/E_6_ Ratio of HLA, C_HLA_, and C_HLA_/C_FLA_ Ratio in the Dark-Brown Residue Obtained from the Polyphenol-Maillard Reaction

**Figure 3:**
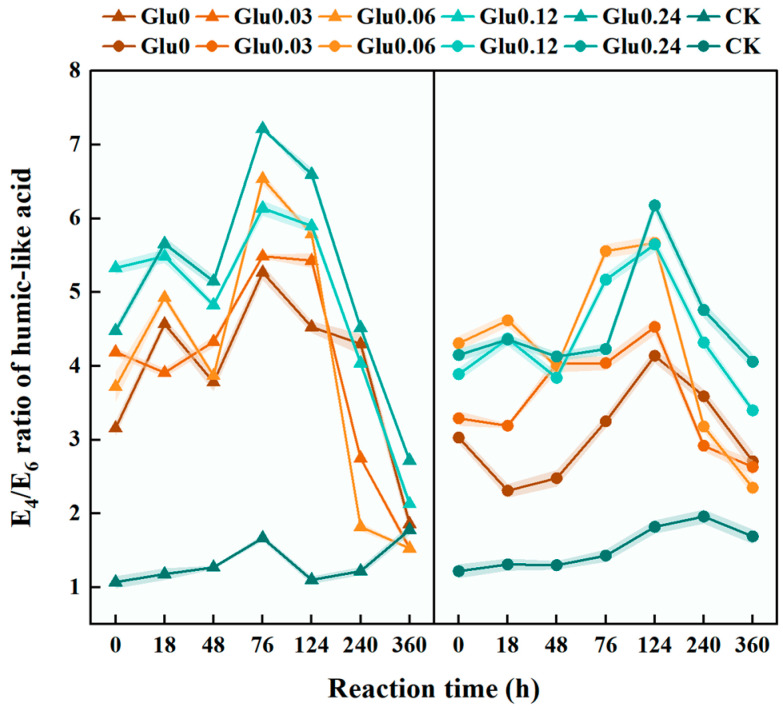
E_4_/E_6_ ratio of HLA extracted from the dark-brown residue generated via the polyphenol-Maillard reaction.

As shown in Figure 3, the E_4_/E_6_ ratio of HLA was a key parameter reflecting the molecular size and degree of aromatic condensation in HLS, where lower values indicated higher molecular weight and greater aromaticity [17]. In the Kao system, the E_4_/E_6_ ratio decreased over the 360 h reaction period for all Glu-amended treatments, with total reductions between 39.4% and 63.4%, reaching a peak at 76 h of reaction. The Glu0.03 treatment exhibited the greatest decline, with a reduction of 63.4%. In the Mon system, the E_4_/E_6_ ratio also decreased across all Glu treatments, with total reductions between 2.2% and 45.6%; the ratio peaked at 124 h before declining, and the Glu0.06 treatment showed the largest reduction at 45.6%. Overall, the decrease in the E_4_/E_6_ ratio was significantly more pronounced in the Kao system than in the corresponding treatments of the Mon system.

**Figure 4:**
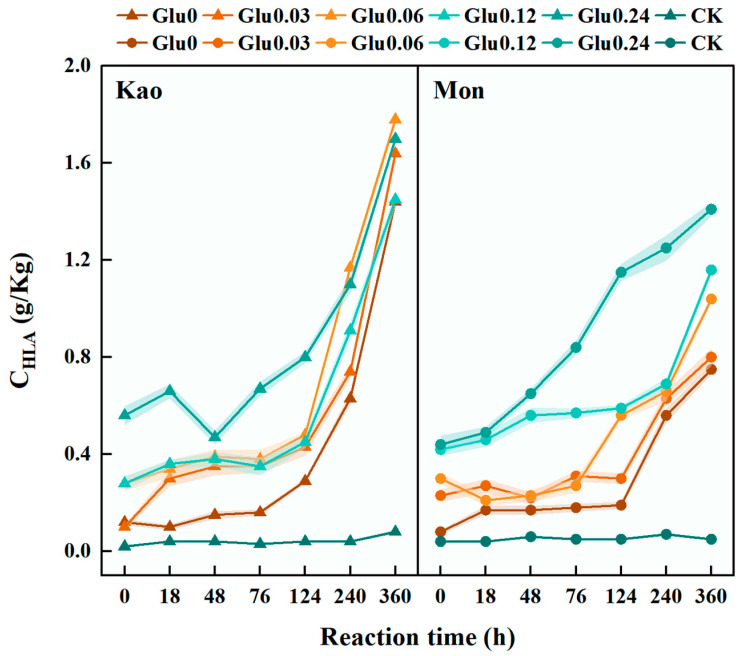
C_HLA_ extracted from the dark-brown residue generated via the polyphenol-Maillard reaction.

As was shown in Figure 4, C_HLA_ was a key indicator for assessing the accumulation of HLA formed during the Maillard reaction [2]. All Glu-amended treatments exhibited significantly higher C_HLA_ content compared to the CK, displaying a general trend of gradual increase over time. In the Kao system, the Glu0.03 treatment showed the highest C_HLA_ growth rate, reaching 1483.5%; in the Mon system, the Glu0.06 treatment achieved the maximum growth rate of 246.1%. Notably, in both systems, the C_HLA_ growth rate in all Glu-amended treatments began to increase significantly starting at 76 h of reaction.

**Figure 5:**
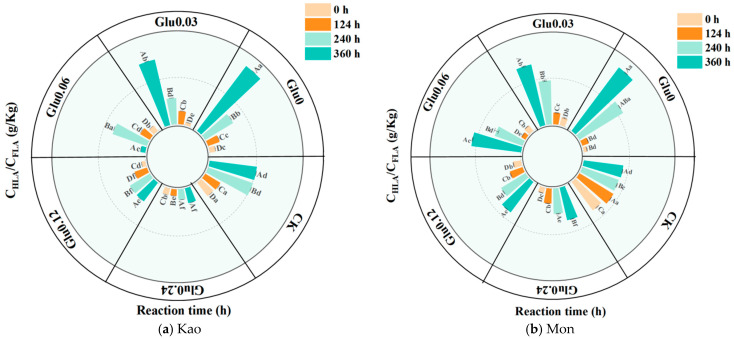
C_HLA_/C_FLA_ ratio in the dark-brown residue generated via the polyphenol-Maillard reaction. Note: Different uppercase letters indicated significant differences (*p* < 0.05) among samples at different incubation times within the same treatment, while different lowercase letters denoted significant differences (*p* < 0.05) across treatments under the same reaction time.

C_HLA_/C_FLA_ ratio was a key parameter for assessing the humification degree and maturity of HLS formed via the Maillard reaction, reflecting the efficiency of the transformation from low-molecular-weight FLA to high-molecular-weight HLA [18]. As was shown in Figure 5, the CK maintained consistently low and stable C_HLA_/C_FLA_ ratios throughout the 360 h reaction period, with initial values of 0.16 and 0.31, and final values of 0.51 and 0.35, respectively. All Glu-amended treatments exhibited a continuous increase in the C_HLA_/C_FLA_ ratio over time. In the Kao system, the Glu0.03 treatment exhibited the highest increase in the C_HLA_/C_FLA_ ratio, reaching a growth rate of 2118.0%; meanwhile, in the Mon system, the Glu0.06 treatment achieved the maximum enhancement, amounting to 699.9%.

### 2.3. FTIR Spectroscopy and Atom Molar Ratios of HLA Derived from Polyphenol-Maillard Reaction

**Figure 6:**
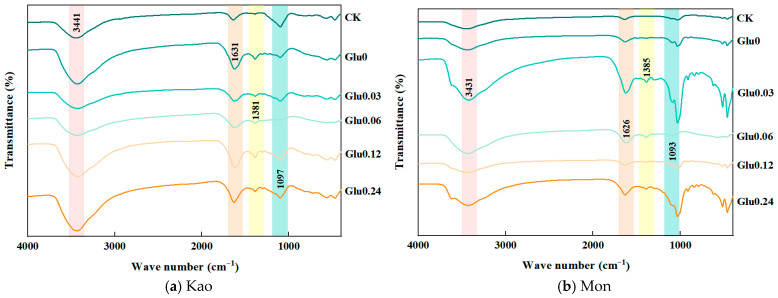
FTIR spectra of HLA extracted from the dark-brown residue generated via the polyphenol-Maillard reaction.

As was shown in Figure 6 and Table 3, the 1086–1109 cm^−1^ region represented the C–O stretching vibration of polysaccharides, directly reflecting the degree of polymerization of the polysaccharide backbone [19]; the 1620–1635 cm^−1^ region represented the C=C stretching vibration of aromatic rings [20] corresponding to the construction of the aromatic ring conjugated system; and the 1385–1394 cm^−1^ region represented the symmetrical bending vibration of methyl C–H, corresponding to the alkyl side chains of the polysaccharide sugar rings or small C-containing products [21]. When exogenous Maillard precursors were added, the absorption peak at 3423–3443 cm^−1^ was consistently greater than that of CK. This was because each exogenous precursor treatment simultaneously exhibited the stretching vibration of –OH or interlayer water molecules of Kao or Mon, as well as the stretching vibration of phenolic hydroxyl and carboxyl hydroxyl groups in HLA. The superposition of these peaks resulted in the peak intensity at 3423–3443 cm^−1^ being greater than that of the CK. The peak intensities at 1086–1109, 1620–1635, and 1385–1394 cm^−1^ all exhibited a trend of first increasing and then decreasing. This indicated that the content of HLA generated by the Maillard reaction did not continuously increase with the increase in Glu concentration, but rather there was an optimal Glu concentration range.

Table 4 presented the effects of varying Glu concentrations on the atom molar ratios (H/C, C/N, and O/C) of HLA in two mineral systems. These ratios reflected key molecular characteristics, including the degree of condensation, abundance of O-containing functional groups, aromaticity, and the relative distribution of C and N [22,23]. The H/C ratio was negatively correlated with aromaticity—lower values indicated a higher extent of aromatic condensation [24]. The C/N ratio reflected the relative abundance of C and N in HLA molecules; reduced C/N values suggested preferential enrichment of N within stable, N-rich structural domains [23]. The O/C ratio served as an indicator of oxidation degree and molecular polarity, with higher values corresponding to greater oxidation and enhanced polarity [23]. The data in Table 4 showed that in the CK, which received no Maillard reaction precursors and contained only (Kao or Mon, no HLA was detected, and thus no elemental composition data were obtained. In the Kao system, compared to the Glu0.03 treatment, the HLA extracted from the residual culture solution in the Glu0.06, Glu0.12, and Glu0.24 treatments exhibited higher H/C ratios and lower O/C ratios. In contrast, in the Mon system, the HLA from the Glu0.06 treatment displayed a lower H/C ratio and a higher O/C ratio. This indicated that in the Kao system, the Glu0.03 treatment most effectively promoted the formation of HLA with elevated aromaticity, whereas in the Mon system, the Glu0.06 treatment favored the development of more oxidized and polar HLA components. Notably, in the treatment with the optimal Glu concentration, the peak intensity at 1620–1635 cm^−1^ (corresponding to aromatic C=C stretching vibration) reached its maximum, which aligned with the relatively lower H/C ratio observed. This finding confirmed enhanced aromatic condensation under these specific concentration conditions. Furthermore, all Glu-amended treatments showed lower C/N ratios than the Glu0 treatment, indicating that Glu addition facilitated the incorporation of N-containing compounds into the HLA structure—a finding was consistent with Wang et al. [2].

### 2.4. FTIR Spectroscopy of Clay Minerals Before and After the Polyphenol-Maillard Reaction

**Figure 7:**
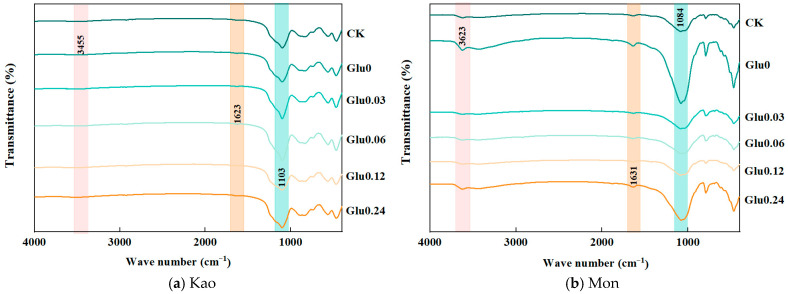
FTIR spectra of clay minerals before and after the polyphenol-Maillard reaction.

As shown in Figure 7 and Table 5, the absorption band at 3421–3473 cm^−1^ was attributed to the stretching vibration of structural O–H in the two clay minerals [25], whereas the band at 1620–1635 cm^−1^ corresponded to the bending vibration of H–O–H in adsorbed water [26]. The band at 1086–1109 cm^−1^ was associated with the Si–O stretching vibration within the Si-O tetrahedra (SiO_4_) [27]. In the Kao system, the low-concentration Glu treatment resulted in lower peak intensities at 3421–3473 cm^−1^ and 1620–1635 cm^−1^ compared to the CK, but higher intensity at 1086–1109 cm^−1^. Conversely, the high-concentration Glu treatment exhibited an opposite pattern. In the Mon system, both low- and high-concentration Glu treatments showed reduced peak intensities at 3421–3473 cm^−1^ and 1620–1635 cm^−1^ relative to the CK, whereas the intensity at 1086–1109 cm^−1^ was consistently enhanced.

## 3. Discussion

### 3.1. Effects of Glu at Different Concentrations on E_4_/E_6_ Ratio and TOC Content in the Supernatant During the Polyphenol-Maillard Reaction

The high R^2^ values of Logistic fitting (>0.8) confirmed that the abiotic humification process followed a typical sigmoidal kinetic pattern, which was characterized by slow initiation, rapid polymerization and stable maturation. During the 360 h reaction period, the E_4_/E_6_ ratio in all Glu-added treatment groups in the Kao and Mon systems consistently showed a downward trend, indicating that the generated HLS underwent progressive polymerization and an increase in the degree of aromatic condensation [28]. This dynamic change could be well fitted by a logistic function, verifying its time-dependent characteristics. The significant increase in *A*_1_ and *A*_2_ parameters further indicated that in the early stage of the Maillard reaction, a higher Glu concentration provided a more abundant C skeleton, promoting the formation of low-molecular-weight intermediates and driving their polymerization towards complex aromatic structures [29]. Notably, the increments of *A*_1_ and *A*_2_ in the Kao system were significantly higher than those in the Mon system. This phenomenon suggested that the Kao system was more conducive to the accumulation of low-molecular-weight intermediates in the early stage of the reaction and promoted the formation of relatively simple high-molecular-weight HLS in the later stage. This might be attributed to the weaker catalytic ability of Kao for aromatization, resulting in a lower conversion efficiency of intermediates to high-aromaticity polymers, thereby causing the accumulation of precursor substances and increasing the *A*_1_ and *A*_2_ values [30]. In contrast, the more significant decrease in the E_4_/E_6_ ratio in the Mon system reflected its stronger catalytic effect on the aromatization process. This difference in catalytic performance could be attributed to the essential differences in surface properties and layered structures between the two clay minerals. Mon had expandable interlayer spaces and a higher cation exchange capacity, which was conducive to the adsorption and enrichment of organic precursors, thereby promoting their catalytic conversion in the interlayer microenvironment [31]. Additionally, the higher surface acidity and larger specific surface area of Mon may have provided more active sites, thus demonstrating superior catalytic efficiency in promoting aromatic condensation reactions [32]. Kao and Mon systems exhibited identical concentration thresholds for Glu treatments across varying concentrations. This convergence suggested that the polyphenol-Maillard reaction-driven abiotic humification had a “self-limiting” characteristic in terms of aromatic polymerization in that once HLS reached a certain level of structural complexity, the polymerization rate slowed down and stabilized [33]. In contrast, the CK group did not converge to the threshold, confirming that exogenous Glu was essential for initiating and completing the humification process leading to mature HLS.

In both mineral systems, *A_min_* and *A_max_* exhibited an upward trend with increasing Glu concentration, indicating that Glu, as a C precursor, significantly enhanced the formation and accumulation of dissolved organic C. This was attributed to the greater availability of substrates at higher Glu concentrations, which promoted Maillard reaction activity and facilitated the generation of HLA and other soluble products [28]. The increases in *A_min_* and *A_max_* were greater in the Kao system than in the Mon system, suggesting that, under the experimental conditions, Kao was more effective than Mon in promoting the conversion of C precursors to dissolved organic C—a finding supported by Xu et al. [34]. Specifically, Kao more effectively suppressed the mineralization of exogenous Glu and induced a negative priming effect, thereby maintaining a relatively higher net soil C balance. From a mineralogical perspective, Kao’s weaker adsorption capacity limited the immobilization of organic C into solid-phase complexes, thus favoring the accumulation of soluble organic C fractions [34]. With respect to the rate parameter *s*, the *s* value was generally higher in the Mon system than in the Kao system, indicating a faster rate of TOC change. This might have been attributed to Mon’s larger specific surface area and higher cation exchange capacity, which enhanced substrate adsorption and interfacial interactions, thereby accelerating reaction kinetics [31]. However, at a Glu concentration of 0.24 mol/L, an inverse trend was observed. At this concentration, the higher *s* value in the Kao system could be attributed to its compact 1:1 layered structure, which was less susceptible to substrate saturation compared to the expandable interlayer structure of Mon [35]. TOC reached its peak at 3 h of reaction and then decreased, which was characterized by the rapid conversion of precursors such as Glu at the initial stage of the reaction, leading to a sharp increase in TOC. In the later stage of the reaction, the reduction in soluble organic C was mainly driven by two processes, the oxidation of a portion of organic C to CO_2_ and its subsequent release, as well as the formation and precipitation of insoluble polymeric substances such as HLS [36].

### 3.2. Effects of Glu at Different Concentrations on E_4_/E_6_ Ratio of HLA, C_HLA,_ and C_HLA_/C_FLA_ Ratio Extracted from the Dark-Brown Residue

In both the Kao and Mon systems, the E_4_/E_6_ ratio continuously decreased in all Glu-amended treatment groups, confirming that Glu promoted the polymerization of HLA [37]. The surface acidity of clay minerals influenced the rate and direction of the Maillard reaction by modulating the pH of the reaction environment [38]. The surface acidity of minerals synergistically optimized the microenvironmental pH in conjunction with organic acids derived from Glu degradation, thereby determining the optimal Glu concentration specific to each mineral system and accounting for the more pronounced reduction in the E_4_/E_6_ ratio observed within the Kao system. Kao exhibited relatively high surface acidity (pH 5.7–6.9) [39], which was well matched with the organic acids produced from the degradation of 0.03 mol/L Glu [40]. These organic acids adjusted the microenvironmental pH to the optimal range for the Maillard reaction, thereby maximizing aromatic polymerization and resulting in the greatest decline in the E_4_/E_6_ ratio. In contrast, Mon had lower surface acidity (pH 7.2) and a strong interlayer cation buffering capacity [41], requiring higher Glu concentrations to generate sufficient organic acids for effective pH modulation. However, its limited pH regulation efficiency resulted in a more moderate decrease in the E_4_/E_6_ ratio compared to the Kao system. The earlier emergence of the E_4_/E_6_ peak in the Kao system was attributed to the rapid dehydration reaction catalyzed by Al–OH sites, which shortened the induction period of the Maillard reaction and accelerated the transformation of small-molecule intermediates [42]. In contrast, the complexation between interlayer cations in Mon and sugar carbonyl groups inhibited the cyclization of Maillard reaction intermediates [43,44], thereby prolonging the initial condensation stage.

The C_HLA_ level in the experimental group was significantly higher than that in the CK, clearly confirming that Glu served as a key precursor for the Maillard reaction-mediated synthesis of HLA [2]. The continuous accumulation of C_HLA_ over time indicated that abiotic humification was fundamentally a progressive polymerization process, with HLA formation and accumulation exhibiting pronounced long-term dynamics [8]. The difference in optimal Glu concentration between the two systems further demonstrated that an appropriate Glu level can significantly enhance the efficiency of HLA synthesis. This finding aligned with Zhu et al. [45], who reported that 5 g/L Glu effectively induces HLA polymerization in Maillard reaction systems, underscoring the critical role of optimal precursor concentration in promoting humification efficiency. Excessive Glu might have inhibited HLA polymerization by competitively occupying active sites on mineral surfaces. Furthermore, the distinct surface acidity and structural properties of the two clay minerals provided a fundamental explanation for the system-dependent differences in optimal Glu concentration. Kao exhibited a 1:1 layered structure with a limited distribution of Al–OH active sites on its surface [46]. According to [28], research on mineral colloid-catalyzed humification indicated that a lower concentration of Glu prevented excessive occupation of Kao’s surface active sites. This ensured sufficient binding space for humification precursors such as Cat and Gly, facilitating their full polymerization through oxidative polymerization and condensation reactions on the Kao surface, thereby enabling efficient accumulation of HLA. In contrast, Mon possessed a 2:1 expandable structure and, in addition to Al–OH sites, was enriched with Mg–OH octahedral sites due to isomorphous substitution [47]. A slightly higher Glu concentration (Glu0.06) was required to activate all catalytic sites, form stable chelates, and establish an efficient reactive microenvironment [48]. Consequently, the optimal Glu concentration for Mon was higher than that for Kao. In both systems, the C_HLA_ growth rate in Glu-amended treatments increased significantly after 76 h of incubation, marking a pivotal shift from a “slow initiation phase” to a “rapid polymerization phase” in the humification process. Prior to 76 h, the reaction primarily involved the initial degradation of Glu and the accumulation of small-molecule intermediates (e.g., aldehydes and ketones) [49], during this stage, intermediate concentrations had not yet reached the critical threshold, resulting in low HLA polymerization rates. At 76 h, accumulated intermediates surpassed the reaction threshold, leading to a marked increase in molecular collision frequency and full activation of catalytic sites on mineral surfaces. These factors synergistically accelerated the polymerization process, culminating in a sharp rise in C_HLA_ growth rate [31,49].

An increase in the C_HLA_/C_FLA_ ratio indicated ongoing condensation reactions, with the progressive enhancement of aromatic C structures representing a hallmark of advanced humification [2]. In this study, the CK received no exogenous C or N precursors, and only trace amounts of naturally occurring organic matter adsorbed onto mineral surfaces were present. This organic matter primarily consisted of low-reactivity compounds—such as stable aliphatic chains—that were unable to supply sufficient active functional groups (e.g., aldehyde, ketone, and amino groups) [50], thereby failing to initiate or sustain the Maillard reaction. All Glu-amended treatments exhibited a continuous rise in the C_HLA_/C_FLA_ ratio throughout the incubation period, confirming that Glu acted as a key precursor by not only promoting the transformation of FLA into HLA but also facilitating the formation of more concentrated and structurally stable humic components [51]. The optimal precursor concentrations differed between the two systems due to their distinct structural characteristics. In the Kao system, Glu at a relatively lower concentration facilitated a more efficient conversion because the dense structure of Kao restricted precursor diffusion, and lower Glu levels mitigated interference [52], thereby enhancing the condensation and cross-linking of C_FLA_ on mineral surfaces. In contrast, the expanded structure of Mon provided a more accessible reaction environment, which necessitated a moderately higher Glu concentration to drive the polymerization and transformation of C_FLA_ [53], ultimately achieving a greater degree of humification maturity.

### 3.3. Effects of Glu at Different Concentrations on FTIR Spectra and Atomic Molar Ratios of HLA Extracted from the Dark-Brown Residue

In the low-concentration Glu stage, the abundant substrate promoted the condensation with amino compounds [54], generating intermediate products such as aldehydes and ketones [55]. These products were polymerized and cyclized to form HLA. Correspondingly, the 1086–1109 cm^−1^ peak increased with the abundance of the polysaccharide backbone, the 1620–1635 cm^−1^ peak increased due to the continuous construction of the aromatic ring conjugated system, and the 1385–1394 cm^−1^ peak increased with the number of alkyl side chains of polysaccharides. When the Glu concentration reached the optimal threshold, the Maillard reaction polymerization efficiency was the highest [56], and the generation of HLA reached its peak. The degree of polymerization of the polysaccharide precursor, the degree of hydration of the system, and the proportion of side chains were all in the optimal state, and the intensities of the three infrared peaks also reached their maximum simultaneously. Excessive Glu diluted the effective concentration of amino compounds [57], inhibited the condensation reaction as a core step, and also promoted non-target side reactions such as Glu self-dehydration [58]. This not only consumed a large amount of reducing sugar but also resulted in small molecules that competed with the HLA precursors for active sites, further hindering polymerization, ultimately leading to a decrease in HLA generation and a simultaneous weakening of the three infrared peak intensities.

The elemental analysis results showed that in the Kao system, the Glu0.03 treatment group had a lower H/C ratio and a higher O/C ratio for the HLA, indicating a higher degree of aromatization and stronger polarity. In contrast, the Glu0.06 treatment group in the Mon system showed the same result. This difference stemmed from the distinct catalytic mechanisms of the two types of clay minerals. The surface of Kao was rich in Lewis acidic sites, which preferentially promoted the dehydration condensation reaction between Glu and amino acids and their degradation products, facilitating the formation of hydrophobic aromatic ring structures and thereby reducing the overall polarity of HLA. This mechanism was highly consistent with the phenomenon observed by Li et al. [8] in the Maillard reaction of Gly-Glu catalyzed by gibbsite, where highly aromatic and strongly hydrophobic HLA was generated. This further confirmed that 1:1 type layered silicates regulated the structural evolution of HLA through a condensation path dominated by surface acid sites. In contrast, Mon, with its 2:1 type expandable layered structure, possessed both Lewis acid and Brønsted acid properties, and could simultaneously promote aromatization and oxidation processes in the Maillard reaction: on the one hand, it accelerated the cyclization and dehydrogenation aromatization of Schiff bases; on the other hand, it activated molecular O or promoted the formation of quinone intermediates, thereby introducing more hydroxyl, carboxyl and other oxygen-containing functional groups, ultimately forming HLA with high aromaticity and high oxidation degree [59]. Additionally, compared with the Glu0 group, the C/N ratio of all Glu-added treatment groups was significantly reduced, indicating that exogenous Glu, as a reducing sugar and a core precursor of humification, could significantly promote the integration of N elements into HLA molecules through the Maillard reaction and condensation with amino acids derived from ammonium N [60]. This phenomenon was attributed to the fact that Glu could act as a polyol precursor to participate in the H transfer catalytic system, generating active alkylating reagents through retroaldol cleavage, providing C sources and reaction driving forces for the construction of N-containing heterocycles and N-alkylated aromatic structures, thereby indirectly increasing the probability of formation of such products [61]. It was worth noting that HLA was not detected in the CK, confirming that the abiotic formation of HLA was dependent on the Maillard reaction triggered by the coexistence of C sources (such as Glu) and N sources (such as amino acids) [2]. Clay minerals themselves did not have the ability to independently initiate and complete the synthesis of humus.

### 3.4. Effects of Glu at Different Concentrations on FTIR Spectra of Clay Minerals Before and After the Polyphenol-Maillard Reaction

Kao possessed Al–OH binding sites [62]. Under low Glu concentration conditions, Cat was adsorbed through coordination and complexation with these Al–OH sites, displacing the previously adsorbed water molecules [63], as illustrated in Equation (3). Notably, Equation (3) could not be balanced by conventional stoichiometric methods because the number of reactive Al–OH sites on the Kao surface was heterogeneous, and the adsorption state of surface water molecules was difficult to quantify with fixed stoichiometric ratios. Concurrently, the coordination interaction of Cat induced a superimposed electron transfer effect [64], leading to a significant increase in the electron cloud density around the Si–O bonds.

(3) $$\mathrm{Kao}–\mathrm{Al}–\mathrm{OH}\cdot H_{2}O\;+\;C_{6}H_{4}(\mathrm{OH}{)}_{2}\;⇌\;\mathrm{Kao}–\mathrm{Al}–O–C_{6}H_{3}(\mathrm{OH})\;+\;H_{2}O$$

The Al–OH sites on the surface of Kao were rich in reactive hydroxyl groups, which could form stable interactions with adsorbates through H bonds or coordination bonds [65]. As the Glu concentration increased, a large number of Glu molecules, with their multi-hydroxyl structure, competitively bound to the Al–OH sites, occupying these active sites and thereby inhibiting the coordination adsorption of Cat [66]. Additionally, the strong hydrophilicity of the hydroxyl groups in Glu molecules promoted the enrichment of free water molecules around them [46], leading to an increase in the intensity of the infrared absorption peak of Kao at 1620–1635 cm^−1^; this change corresponded to the gradual recovery of the electron cloud density of the Si–O bond, approaching the initial level, as shown in Equation (4). Notably, Equation (4) could not be balanced by conventional stoichiometric methods, because the H and O atoms involved in the reaction were dynamically compensated by the lattice hydroxyl groups on the mineral surface and free water in the system. In the system, parameter *n* represented the initial free water content, while parameter *x* denoted the amount of water enriched by the hydrophilic interaction with Glu. It was noteworthy that Kao, as a 1:1-type layered clay mineral with a compact structure, enabled effective adsorption of HLS derived from the abiotic humification of phenolic compounds, Gly, and Glu acid on its surface [45,66]. These HLS were rich in multi-hydroxyl functional groups, further enhancing the hydrophilicity of the mineral surface [67]. Therefore, under high-concentration Glu treatment conditions, the intensity of the infrared absorption peak at 1620–1635 cm^−1^ was significantly higher than that of the CK.

(4) $$\mathrm{Kao}–\mathrm{Al}–O–C_{6}H_{3}(\mathrm{OH})+{\;C}_{6}H_{2}O_{6}\;+\;nH_{2}O\;⇌\;\mathrm{Kao}–\mathrm{Al}–\mathrm{OH}\cdot C_{6}H_{2}O_{6}\;+\;C_{6}H_{4}(\mathrm{OH}{)}_{2}\;+\;(n\;+\;x)H_{2}O$$

Due to isomorphic substitution, some Al^3+^ in Mon was replaced by Mg^2+^ with a similar radius but a lower charge, thus containing Mg–OH binding sites [47]. Under low Glu concentration conditions, Cat not only formed coordination complexes with Al–OH sites but also formed chelate structures with Mg–OH sites [63,68], resulting in a significant decrease in the intensity of absorption peaks in the range of 3421–3473 cm^−1^ and 1620–1635 cm^−1^, as shown in Equation (5). Notably, Equation (5) could not be balanced by conventional stoichiometric methods because the number of reactive Al–OH and Mg–OH sites on the Mon surface was heterogeneous and dependent on the degree of isomorphic substitution, and lattice oxygen atoms from the mineral matrix also participated in the chelation reaction. Due to the superimposition of the multiple surface binding effects of Cat on both Al–OH and Mg–OH sites, the transfer of electrons to the siloxane tetrahedral framework was more significant, leading to a greater increase in the intensity of the Si–O bond vibration peak than in the Kao system [69].

(5) $$\mathrm{Mon}–\mathrm{Mg}–\mathrm{OH}\;+\;\mathrm{Mon}–\mathrm{Al}–\mathrm{OH}\;+\;C_{6}H_{4}(\mathrm{OH}{)}_{2}\;⇌\;\mathrm{Mon}–\mathrm{Mg}–O–C_{6}H_{3}(\mathrm{OH})\;+\;\mathrm{Mon}–\mathrm{Al}–O–C_{6}H_{3}(\mathrm{OH})+H_{2}O\;$$

Under high Glu concentration conditions, Mon was a 2:1 type layered clay mineral with a relatively large interlayer space [70]. However, the HLS generated through abiotic humification could only be adsorbed on its surface [71]. The hydroxyl and carbonyl groups on its surface could form weak H bonds with the functional groups in organic chelates [72], further stabilizing the complex structure. Therefore, only the Al–OH sites were mainly competed by high Glu concentration, while the Mg–OH sites remained unoccupied [73], and the adsorbed water could not be fully restored, resulting in the peak intensities at 3421–3473 cm^−1^ and 1620–1635 cm^−1^ remaining persistently lower than those of the CK. At all Glu concentrations, the intensity of the Si–O peak in Mon consistently exceeded that in CK. This phenomenon was attributed to electron cloud modulation resulting from the stable chelation of Cat at Mg–OH sites, which enhanced the vibration of Si–O tetrahedra. The associated processes were described by Equations (6) and (7). Notably, Equations (6) and (7) could not be balanced by conventional stoichiometric methods, because the adsorption and chelation processes involved heterogeneous interactions between organic molecules and mineral surfaces, and the participation of surface functional groups (e.g., hydroxyl, carbonyl) and lattice oxygen was dynamic and difficult to quantify with fixed stoichiometric ratios.

(6) $$\mathrm{Mon}–\mathrm{Mg}–O–C_{6}H_{3}(\mathrm{OH})\;+{\;C}_{6}H_{12}O_{6}\;\to \;\mathrm{No}\;\mathrm{response}$$

(7) $$\mathrm{Mon}–\mathrm{Al}–O–C_{6}H_{3}(\mathrm{OH})\;+\;C_{6}H_{12}O_{6}\;+\;nH_{2}O\;⇌\;\mathrm{Mon}–\mathrm{Al}–\mathrm{OH}\cdot C_{6}H_{12}O_{6}\;+\;C_{6}H_{4}(\mathrm{OH}{)}_{2}\;+\;(n\;+\;x)H_{2}O$$

## 4. Materials and Methods

### 4.1. Materials

Pure standards of Kao, Mon, Cat, Glu and Gly were all purchased from Shanghai Chemical Reagent Co., Ltd., Shanghai, China, a subsidiary of China National Pharmaceutical Group Corporation.

Key properties of the two minerals at pH = 8 were determined experimentally. Kao had a CEC of 8.2 cmol/kg, specific surface area of 15.6 m^2^/g, and surface charge density of 0.04 C/m^2^; Mon had a CEC of 112.5 cmol/kg, specific surface area of 756.3 m^2^/g, and surface charge density of 0.02 C/m^2^.

Preparation of 0.2 mol/L phosphate-buffered solution (pH 8.0): Two stock solutions were prepared, respectively—31.2 g of NaH_2_PO_4_·2H_2_O was weighed and dissolved in 1000 mL of distilled water to prepare a 0.2 mol/L NaH_2_PO_4_ stock solution; 71.632 g of Na_2_HPO_4_·12H_2_O was weighed and dissolved in 1000 mL of distilled water to prepare a 0.2 mol/L Na_2_HPO_4_ stock solution. Then, 5.3 mL of the 0.2 mol/L NaH_2_PO_4_ stock solution was mixed thoroughly with 94.7 mL of the 0.2 mol/L Na_2_HPO_4_ stock solution, and finally 0.02% (*w*/*v*) thimerosal was added as a preservative to obtain the target buffer solution.

### 4.2. Experimental Design and Determination of Chemical Properties

All experiments were conducted under strictly sterile conditions to ensure that the abiotic transformation process dominated. Prior to use, all glassware and the phosphate-buffered solution (0.2 mol/L) were sterilized by autoclaving at 121 °C for 20 min. A series of 500 mL conical flasks was prepared for each treatment: each flask received 250 mL of the sterilized phosphate-buffered solution and 2 g of either Kao or Mon. Cat and Gly were added to each flask to achieve a final concentration of 0.06 mol/L each. A Glu concentration gradient was established across treatments: 0, 0.03, 0.06, 0.12, and 0.24 mol/L. Corresponding treatment groups were designated Glu0, Glu0.03, Glu0.06, Glu0.12, and Glu0.24. The CK consisted solely of 250 mL of 0.2 mol/L phosphate-buffered solution and 2 g of Kao or Mon—without Glu, Cat, or Gly—and was labeled CK. All treatments, including the control, were performed in triplicate to ensure experimental reproducibility.

Liquid-phase shake-flask incubations were conducted under aseptic conditions at 150 rpm. Samples were collected from the 500 mL conical flasks at predetermined time points (0, 3, 6, 18, 28, 48, 76, 124, 172, 240, and 360 h). Each sample was then subjected to high-speed centrifugation (16,000× *g*, 15 min) to separate the supernatant from the dark-brown residue formed via the polyphenol–Maillard reaction. 2 mL aliquots of supernatant were withdrawn from each replicate system. All collected samples were immediately centrifuged at 16,000× *g* for 15 min to remove residual particulates. Thereafter, 1 mL of the clarified supernatant was diluted to 25 mL with deionized water, and absorbances at 465 nm (E_4_) and 665 nm (E_6_) were measured using a UV-visible spectrophotometer (TU-1900, Beijing Purkinje General Instrument Co., Ltd., Beijing, China). The E_4_/E_6_ ratio—widely recognized as an empirical indicator of aromatic condensation extent and humification degree in aqueous organic matter—was calculated accordingly. Concurrently, parallel supernatant subsamples were analyzed for TOC content using a Vario TOC cube analyzer (Elementar Analysensysteme GmbH, Hanau, Germany). In parallel, 10 mL aliquots of supernatant were collected at identical time intervals and acidified to pH 1.0 with 1.0 mol/L HCl. Following 24 h equilibration at room temperature, samples were centrifuged (16,000× *g*, 10 min). The resulting acidic supernatant was designated as the FLA fraction and subsequently neutralized (to pH ≈ 7.0) prior to dilution and analysis. The pellet was defined as the HLA fraction; it was solubilized in 0.1 mol/L NaOH, neutralized, and diluted to yield a standardized HLA solution. The E_4_/E_6_ ratio of this HLA solution was determined identically to that of the original supernatant. Both FLA and HLA fractions were quantified for C content (C_FLA_ and C_HLA_, respectively) via TOC analysis, and the C_HLA_/C_FLA_ ratio was computed as an index of humification progression. At the conclusion of the 360 h incubation, bulk HLA was recovered from the residual incubation medium by acid precipitation (pH 1.0). The precipitate was redissolved in 0.1 mol/L NaOH and subjected to demineralization using a 6% (*v*/*v*) HCl–6% (*v*/*v*) HF mixture. After centrifugation (16,000× *g*, 10 min), the purified HLA solution was processed by electrodialysis, followed by lyophilization. The dried HLA solid was ground and sieved to obtain a homogeneous powder with particle size ≤ 0.01 mm, suitable for subsequent characterization. C, H, O, and N contents in the solid HLA samples were determined via elemental analysis using a CHNS/O analyzer (PerkinElmer 2400 Series II, Waltham, MA, USA). Structural characteristics of the purified HLA powder were characterized by FTIR spectroscopy (model FTIR-850, Tianjin Gangdong Science and Technology Development Co., Ltd., Tianjin, China). To minimize interference from moisture and ensure spectral accuracy, FTIR spectroscopy were recorded under optimized conditions: resolution of 4 cm^−1^, scanning range of 400–4000 cm^−1^, 32 cumulative scans, and measurements conducted in a nitrogen atmosphere (humidity < 5%) to avoid interference from atmospheric moisture. Prior to pellet pressing, samples were prepared via the KBr pellet method with a sample-to-KBr mass ratio of 1:100, and vacuum-dried at 60 °C for 24 h to thoroughly remove adsorbed water. Spectral data were acquired and processed using the proprietary FTIR-850 2.0.3 software. A 0.2 mol/L phosphate-buffered solution (pH = 8.0) was used to maintain system stability, and its buffer capacity was verified by pre-experiments to offset the effects of organic acids produced by Glu degradation.

The pH of the system was monitored every 48 h during the experiment, and the results showed that the pH fluctuation range of all treatment groups was 7.8–8.2, with no significant local acidification. Meanwhile, the pH of the supernatant was measured with a pH meter (accuracy ± 0.01) every 48 h to ensure local pH changes did not exceed 0.5 units, avoiding unintended dissolution of aluminosilicates.

### 4.3. Statistical Analyses

The experimental data were visualized using Origin 2021 software, and statistical analyses were performed with SPSS 18.0. Intergroup differences were assessed via one-way analysis of variance (ANOVA), followed by post hoc comparisons using the least significant difference (LSD) test to determine statistical significance.

## 5. Conclusions

Montmorillonite, with its expandable interlayer structure, high cation exchange capacity and Mg–OH binding sites, had a significantly stronger catalytic ability for the aromatization polymerization and structural stabilization of HLA than kaolinite. Kaolinite, due to its 1:1 type dense structure and Al–OH binding site characteristics, was more conducive to the accumulation of soluble organic C and the retention of early low-molecular-weight intermediates. The formation of HLS exhibited optimal glucose concentration ranges: 0.03 mol/L for kaolinite and 0.06 mol/L for montmorillonite, rather than increasing continuously with elevated glucose concentrations. At these concentrations, the accumulation efficiency and structural maturity of HLA were the highest. The FTIR spectra confirmed that the chelate formed by montmorillonite and the precursors were more stable and could maintain structural stability even at high glucose concentrations. However, the active sites on the surface of kaolinite were easily occupied by high-concentration glucose, and its regulation of HLA was more susceptible to the influence of glucose concentration.

## Figures and Tables

**Table 1:** Logistic function fitting results for E_4_/E_6_ ratio in the supernatant.

Mineral Types	Treatments	Fitting Equation (1) to the E_4_/E_6_ Ratio $y_{1}\;=\;[A_{2}\;+\;(A_{1\;}-\;A_{2})]/[1\;+\;(x/x_{0}{)}^{p}]$	Coefficient
Kao	Glu0	y_1_ = 2.57688 + (3.28823 − 2.57688)/[1 + (x/7.59304)^0.34405^]	R^2^ = 0.99902
	Glu0.03	y_1_ = 2.77323 + (7.49808 − 2.77323)/[1 + (x/7.52196)^0.57146^]	R^2^ = 0.99937
	Glu0.06	y_1_ = 4.23026 + (9.56222 − 4.23026)/[1 + (x/3.94001)^1.04831^]	R^2^ = 0.99835
	Glu0.12	y_1_ = 5.4871 + (11.85907 − 5.4871)/[1 + (x/7.48577)^0.94353^]	R^2^ = 0.99866
	Glu0.24	y_1_ = 7.45618 + (18.22455 − 7.45618)/[1 + (x/3.60418)^0.63583^]	R^2^ = 0.99722
	CK	y_1_ = 0.30579 + (1.28813 − 0.30579)/[1 + (x/110.03966)^1.14851^]	R^2^ = 0.99968
Mon	Glu0	y_1_ = 2.25521 + (4.99665 − 2.25521)/[1 + (x/9.79164)^0.49331^]	R^2^ = 0.97283
	Glu0.03	y_1_ = 3.16625 + (5.89474 − 3.16625)/[1 + (x/0.83661)^0.39197^]	R^2^ = 0.99998
	Glu0.06	y_1_ = 4.39492 + (9.4031 − 4.39492)/[1 + (x/2.31909)^0.60423^]	R^2^ = 0.99886
	Glu0.12	y_1_ = 4.43706 + (12.78133 − 4.43706)/[1 + (x/10.81465)^0.84675^]	R^2^ = 0.99687
	Glu0.24	y_1_ = 5.67417 + (16.56004 − 5.67417)/[1 + (x)13.84915)^1.0157^]	R^2^ = 0.99864
	CK	y_1_ = 1.87281 + (1.00594 − 1.87281)/[1 + (x/26.75154)^0.8829^]	R^2^ = 0.99906

Note: A gradient of Glu concentrations—namely, 0, 0.03, 0.06, 0.12, and 0.24 mol/L—was designated as Glu0, Glu0.03, Glu0.06, Glu0.12, and Glu0.24, respectively. The CK comprised 250 mL of 0.2 mol/L phosphate-buffered solution supplemented with 2 g of Kao or Mon, and contained no added Glu, Cat, or Gly. This convention was the same as below. Treatments with Glu concentrations of 0.03 and 0.06 mol/L were classified as low-concentration conditions; those at 0.12 and 0.24 mol/L were classified as high-concentration conditions. This convention was the same as below.

**Table 2:** Logistic function fitting results for TOC content in the supernatant generated via the polyphenol-Maillard reaction.

Mineral Types	Treatments	Fitting Equation (2) to the TOC Content $y_{2}\;={\;A}_{\min}\;+\;(A_{\max}-{\;A}_{\min})/[1\;+\;(x_{0}/x{)}^{h}{]}^{s}$	Coefficient
Kao	Glu0	y_2_ = 4.3517 + (7.8902 − 4.3517)/[1 + (92.34078/x)^−0.01508^]^1^	R^2^ = 0.99978
	Glu0.03	y_2_ = 6.8799 + (11.0169 − 6.8799)/[1 + (7.05579/x)^0.06766^]^0.14124^	R^2^ = 0.99743
	Glu0.06	y_2_ = 10.1239 + (11.33604 − 10.1239)/[1 + (14.15495/x)^−11.71588^]^0.05699^	R^2^ = 0.99934
	Glu0.12	y_2_ = 11.4183 + (21.5699 − 11.4183)/[1 + (2863.07929/x)^5.44322^]^0.04476^	R^2^ = 0.99832
	Glu0.24	y_2_ = 20.8921 + (31.3766 − 20.8921)/[1 + (3.54692/x)^−0.42647^]^1.28719^	R^2^ = 0.98785
	CK	y_2_ = 0.67667 + (0.94594 − 0.67667)/[1 + (275.07156/x)^−0.08384^]^0.97606^	R^2^ = 0.99403
Mon	Glu0	y_2_ = 5.8942 + (7.75833 − 5.8942)/[1 + (16.79984/x)^−0.61978^]^1.37788^	R^2^ = 0.99703
	Glu0.03	y_2_ = 5.64105 + (9.9841 − 5.64105)/[1 + (9.81865/x)^−0.19256^]^1.43169^	R^2^ = 0.98490
	Glu0.06	y_2_ = 7.5719 + (13.4927 − 7.5719)/[1 + (306.99547/x)^−0.07321^]^1.07976^	R^2^ = 0.87627
	Glu0.12	y_2_ = 12.4036 + (20.5827 − 12.4036)/[1 + (58.57826/x)^−0.42565^]^1.39099^	R^2^ = 0.90699
	Glu0.24	y_2_ = 21.56114 + (26.85043 − 21.56114)/[1 + (39.07596/x)^−0.05134^]^1.15424^	R^2^ = 0.80175
	CK	y_2_ = 0.5822 + (1.3607 − 0.5822)/[1 + (141.87862/x)^−1.88495^]^189.85594^	R^2^ = 0.99933

**Table 3:** FTIR Spectroscopy relative intensities (% of total area) of HLA extracted from the dark-brown residue generated via the polyphenol-Maillard reaction, as influenced by Glu concentration in the presence of Kao or Mon.

Mineral Types	Treatments	Peak Position of FTIR Characteristic Absorption Peak (cm^−1^)			
		3423–3443 Stretching Vibration of –OH	1620–1635 C=C Stretching Vibration of Aromatic Rings	1385–1394 Symmetrical Bending Vibration of Methyl C–H	1086–1109 C–O Stretching Vibration of Polysaccharides
Kao	Glu0	79.0 ± 0.5 c	14.0 ± 0.4 a	1.5 ± 0.3 c	5.5 ± 0.1 d
	Glu0.03	76.6 ± 0.4 e	14.1 ± 0.4 a	2.3 ± 0.2 a	7.1 ± 0.2 b
	Glu0.06	80.0 ± 0.6 b	13.6 ± 0.5 b	1.4 ± 0.1 c	5.0 ± 0.1 e
	Glu0.12	78.4 ± 0.2 d	13.1 ± 0.2 c	1.8 ± 0.2 b	6.8 ± 0.2 c
	Glu0.24	81.3 ± 0.6 a	9.9 ± 0.2 d	1.9 ± 0.1 b	6.9 ± 0.1 c
	CK	76.0 ± 0.3 f	6.0 ± 0.1 e	1.5 ± 0.4 c	16.5 ± 0.4 a
Mon	Glu0	88.6 ± 0.7 a	9.7 ± 0.2 f	0.4 ± 0.2 d	1.3 ± 0.2 d
	Glu0.03	86.6 ± 0.4 d	11.4 ± 0.2 c	0.8 ± 0.2 c	1.3 ± 0.2 d
	Glu0.06	82.8 ± 0.2 e	12.8 ± 0.4 b	1.5 ± 0.1 a	3.0 ± 0.1 a
	Glu0.12	87.7 ± 0.3 b	10.4 ± 0.4 e	0.4 ± 0.1 d	1.5 ± 0.1 c
	Glu0.24	87.1 ± 0.5 c	11.1 ± 0.4 d	1.1 ± 0.2 b	0.6 ± 0.2 e
	CK	81.8 ± 0.4 f	14.5 ± 0.2 a	1.1 ± 0.3 b	2.5 ± 0.3 b

Note: Different lowercase letters denoted significant differences among treatments, determined by one-way analysis of variance (ANOVA) followed by the least significant difference (LSD) test (*p* < 0.05). This convention was the same as below. The absorption peak observed at 1620–1635 cm^−1^ was exclusively attributed to the aromatic ring system. In the table, distinct lowercase letters indicated the statistical significance of mean differences between groups: groups marked with the same letter did not differ significantly, while those bearing different letters did. Letters were assigned in descending order of means, starting with the group with the highest mean labeled “a”. Groups that did not show significant differences from it were also labeled “a”. A change to the next letter (e.g., “b”) occured when a significant difference was detected, and this pattern continued accordingly. The same convention applied to Table 4 and Table 5.

**Table 4:** Atom molar ratios of HLA extracted from the dark-brown residue generated via the polyphenol-Maillard reaction.

Mineral Types	Treatments	H/C Ratio	C/N Ratio	O/C Ratio
Kao	Glu0	0.97 ± 0.03 c	16.19 ± 0.02 b	0.59 ± 0.05 d
	Glu0.03	0.95 ± 0.07 c	13.48 ± 0.02 d	0.84 ± 0.04 cd
	Glu0.06	1.03 ± 0.03 ab	15.25 ± 0.02 b	0.69 ± 0.03 bc
	Glu0.12	1.08 ± 0.03 a	14.87 ± 0.03 c	0.75 ± 0.03 b
	Glu0.24	1.07 ± 0.03 a	15.57 ± 0.07 a	0.79 ± 0.05 a
Mon	Glu0	1.02 ± 0.03 d	21.25 ± 0.02 a	1.00 ± 0.02 b
	Glu0.03	1.31 ± 0.04 c	16.68 ± 0.03 d	0.85 ± 0.03 d
	Glu0.06	1.00 ± 0.02 d	17.68 ± 0.03 b	1.26 ± 0.02 a
	Glu0.12	1.69 ± 0.02 a	15.03 ± 0.02 e	0.87 ± 0.03 cd
	Glu0.24	1.58 ± 0.03 b	17.54 ± 0.05 c	0.92 ± 0.05 c

**Table 5:** FTIR Spectroscopy relative intensities (% of total area) of clay minerals before and after polyphenol-Maillard reaction.

Mineral Types	Treatments	Peak Position of FTIR Characteristic Absorption Peak (cm^−1^)		
		3421–3473 Stretching Vibration of O–H	1620–1635 Bending Vibration of H–O–H in Adsorbed Water	1086–1109 Si–O Stretching Vibration Within the Si–O Tetrahedra (SiO_4_)
Kao	Glu0	8.1 ± 0.3 d	0.6 ± 0.2 a	91.3 ± 0.2 c
	Glu0.03	5.4 ± 0.1 e	0.3 ± 0.4 b	94.3 ± 0.1 b
	Glu0.06	3.6 ± 0.4 f	0.3 ± 0.1 b	96.1 ± 0.2 a
	Glu0.12	9.4 ± 0.3 a	0.7 ± 0.2 a	89.9 ± 0.5 f
	Glu0.24	9.1 ± 0.1 b	0.8 ± 0.3 a	90.2 ± 0.1 e
	CK	8.3 ± 0.2 c	0.6 ± 0.3 a	91.1 ± 0.4 d
Mon	Glu0	19.2 ± 0.3 b	1.2 ± 0.5 d	79.7 ± 0.2 e
	Glu0.03	14.7 ± 0.2 f	1.5 ± 0.3 c	83.8 ± 0.3 a
	Glu0.06	17.0 ± 0.4 c	1.6 ± 0.2 c	81.3 ± 0.1 d
	Glu0.12	16.4 ± 0.1 e	1.9 ± 0.4 b	81.7 ± 0.5 c
	Glu0.24	16.7 ± 0.1 d	2.0 ± 0.1 b	82.7 ± 0.2 b
	CK	20.8 ± 0.4 a	2.6 ± 0.2 a	76.7 ± 0.3 f

Note: Glu0, Glu0.03, Glu0.06, Glu0.12, and Glu0.24 represented Kao or Mon samples subjected to the polyphenol-Maillard reaction followed by H_2_O_2_ rinsing; CK denoted the corresponding blank control minerals, which underwent identical H_2_O_2_ rinsing but were not exposed to the polyphenol-Maillard reaction. The absorption peak at 1620–1635 cm^−1^ corresponded to the bending vibration of H–O–H in water adsorbed by clay minerals.

## Data Availability

Data will be made available on request.

## Abbreviations

The following abbreviations are used in this manuscript:

Soil organic matter	SOM
Total organic carbon	TOC
Glucose	Glu
Catechol	Cat
Glycine	Gly
Humic-like substances	HLS
Kaolinite	Kao
Montmorillonite	Mon
Humic-like acid	HLA
Fulvic-like acid	FLA
Fourier-transform infrared spectroscopy	FTIR spectroscopy
Cation exchange capacity	CEC
